# The Pyrolysis of (−)-(S)-Nicotine: Racemization and Decomposition

**DOI:** 10.1002/chir.20760

**Published:** 2009-07-30

**Authors:** Peter Clayton, Annhelen Lu, Louise Bishop

**Affiliations:** Group R&D Centre, British American TobaccoSouthampton, United Kingdom

**Keywords:** pyrolysis, oxidative pyrolysis, (−)-(S)-nicotine, nicotine enantiomers, racemization, thermal decomposition, β-nicotyrine

## Abstract

The pyrolytic behaviour of (−)-(S)-nicotine in methanol was investigated using on-line pyrolysis GC/MS to establish whether racemization to the R(+) antipode occurs and to identify other products of pyrolysis. The conditions used included pyrolysing the sample for 15 seconds in an atmosphere of 9% oxygen in nitrogen (275ml/min total flow) across the temperature range of 200°C–1000°C. A chiral Cyclodex-B analytical column (30m × 0.25mm i.d. × 0.25 μm film thickness) was used to separate the enantiomers of nicotine, although the two enantiomer peaks were not baseline resolved. The results of the experiment shows that there is no increase in (+)-(R)-nicotine levels across a wide temperature range. This suggests that the elevated levels of (+)-R-nicotine observed in tobacco smoke (compared to tobacco leaf material) are not due to the pyrolytic auto-racemization of (−)-(S)-nicotine but are a result of more complex interactions between (−)-(S)-nicotine and other smoke components. The pyrolysis of isotopically labelled nicotine established that nicotine undergoes thermal decomposition to β-nicotyrine which in turn may decompose to other products. Chirality 2010. © 2009 Wiley-Liss, Inc.

## INTRODUCTION

**Fig. 1:**
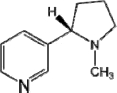
Molecular structure of (−)-(S)-nicotine.

Stereochemical configuration has an important effect on the actions and potency of nicotine in biological systems.^1^ (−)-(S)-nicotine (Fig. 1), the dominant alkaloid in tobacco, is an agonist at neuronal nicotinic acetylcholine receptor subtypes and is considerably more potent than the (+)-(R) antipode.

This work was aimed at examining the pyrolytic behavior of (−)-(S)-nicotine; pyrolysis experiments are commonly used to establish the relationship between tobacco components and products found in smoke.^2^ In particular, the formation of (+)-(R)-nicotine and other possible decomposition products originating from (−)-(S)-nicotine were investigated. The pyrolytic behavior of pure (−)-(S)-nicotine is of interest because it is a step toward a full understanding of the behavior of (−)-(S)-nicotine in a burning cigarette.

It has been observed that, in the tobacco plant, nicotine is overwhelmingly synthesized as (−)-(S)-nicotine and the (+)-(R)-nicotine isomer is present only in minute amounts. (+)-(R)-Nicotine amounts to only 0.2–1% of the total nicotine in raw and processed tobacco, whereas in tobacco smoke the proportion of (+)-(R)-nicotine relative to total nicotine is higher (2–3%).^3^–^5^ The mechanism of the formation of (+)-(R)-nicotine during smoking has been open to speculation. One mechanism advanced is that the elevated temperature encountered during smoking causes nicotine to undergo partial racemization. This hypothesis has recently received support from Liu et al.,^6^ who concluded that the racemization of nicotine is temperature-dependent in both tobacco and pure nicotine standard, with the ratio of (+)-(R)-nicotine to total nicotine reported to be as high as 21.8% following the elevation of (−)-(S)-nicotine to 5508C. This mechanism could be termed the temperature-induced racemization of nicotine. Another mechanism advanced for the occurrence of (+)-(R)-nicotine found in smoke is that (+)-(R)-nicotine could be formed from the radical methylation of nornicotine. Unlike nicotine, norni-cotine is present in tobacco in both R and S configurations^7^ and so methylation will yield both enantiomers of nicotine. However, for this to occur the flux of methyl radicals produced in smoke needs to be considerable (Perfetti, personal communication). A third hypothesis to account for the (+)-(R)-nicotine present in smoke, is that an unknown component in cigarette smoke induces or promotes the partial racemization of (−)-(S)-nicotine. If the second or third mechanism accounts for all the (+)-(R)-nicotine presence in smoke, then heating a sample of pure (−)-(S)-nicotine will fail to produce the (+)-(R) antipode. We now present an investigation into whether (−)-(S)-nic-otine is able to undergo autoracemization under the agency of heat and also to identify other products that are formed from the thermal decomposition of (−)-(S)-nico-tine.

The experimental conditions were chosen to simulate as much as possible the conditions inside a burning cigarette.^2^ Nicotine standard samples were pyrolysed for 15 sec at temperatures in the range of 200–1000°C in an atmosphere of 9% oxygen in nitrogen flow. Following this, the products of pyrolysis were directly introduced on to a chiral GC column and detected by mass spectrometry— that is pyrolysis was online with GC separation and MS detection.

## EXPERIMENTAL

### Preparation of(−)-(S)-Nicotine Standards Sample

(−)-(S)-Nicotine and racemic nicotine were purchased from Fluka (Dorset, UK) (declared chemical purity 99.0%). The enantiomeric purity of (−)-(S)-nicotine was measured by ourselves with the pyrolysis temperature of the pyrolysis unit set at 200°C. The temperature of the inlet was 240°C (at maximum), see Instrumentation and Injection of Samples for full details. It was found that the enantiomeric purity of the (−)-(S)-nicotine standard used in these experiments was 97-98%. (2,4,5,6)-d_4_-R/S-nicotine was purchased from Toronto Research Chemicals (Toronto, Canada). Methanolic solutions of 1.0 mg/ml were prepared from these compounds typically by accurately weighing out 15–20 mg into a 20 ml borosilicate screw top vial and adding by pipette the appropriate volume of HPLC grade methanol (Rathburn Chemicals, Walkerburn, UK) to produce a 1.0 mg/ml solution. Solutions were stored at +4°C. No increase in the level of (+)-(R)-nicotine in 1.0 mg/ml methanol solution of (−)-(S)-nicotine was observed even after storage for over 1 mo.

### Instrumentation and Injection of Samples

Prepared standard solutions were injected into the py-rolysis-GC/MS instrument for pyrolysis, GC separation, and MS detection. A 10 ll aliquot of the prepared (−)-(S)-nicotine (1.0 mg/ml) solution was injected onto a piece of quartz wool (∼2-mm long, used once only) that was placed beforehand inside a quartz tube (2 mm i.d., 25-mm long), using a 10 μL (± 1%) SGE manual microsyringe. The quartz tube was then placed inside the pyroprobe, which was subsequently inserted into a CDS 2000 Pyrolyser module (CDS Analytical, PA) where thermal decomposition was carried out at defined temperatures. The pyrolyser chamber was directly interfaced with an Agilent 6890 gas chro-matograph. The products of pyrolysis were separated on a chiral capillary GC column (Chiral Cyclodex-B, 30 m length × 0.25 μm i.d. × 0.25 μm film thickness, 10.5% β-cyclodextrin in DB-1701 from Agilent Technologies). Mass spectrometric detection was carried out using an Agilent 5973 Mass Spectrometer detector and peaks were identified by the Wiley library of mass spectra. Pyrolysis conditions were 9% oxygen in nitrogen and a total flow rate of 275 ml/min. Samples were pyrolysed for 15 sec at each of the following temperatures: 200, 300, 400, 500, 600, 800, and 1000°C. The carrier gas used to transfer the pyrolysis products to the GC inlet was helium. To prevent condensation of pyrolysis products in the pyolyser-GC interface, the connecting capillary tube was maintained at a constant temperature of 300°C, apart from the pyrolysis experiment at 200°C in which the interface tube was also set to 2008 C. The initial temperature of the GC inlet was set at −60°C, using liquid nitrogen. Following the injection of a sample, the temperature of the inlet was held at −60°C for 1 min. The temperature of the inlet was then ramped up to 240°C at 12°C/sec (25 sec).

The initial temperature of the GC oven was set at 70°C and following injection this was held for 2 min. The temperature was then ramped to 110°C with a temperature gradient of 0.5°C/min. Following this, the temperature gradient was increased to 50°C/min until a final temperature of 220°C was attained, where it was held for 30 min. The GC inlet split ratio was 50:1; the column was kept at a constant flow of helium at 1.5 ml/min and at a pressure of 14.05 psi. The mass spectrometer detector was set to scan mass fragments from 33 to 400 amu.

Each pyrolysis temperature was completed in triplicate; in between each pyrolysis run, a new piece of quartz wool was put into the quartz tube and analyzed as a blank, to eliminate possible carryover between samples. If carryover was observed, as occasionally was observed at the lower temperatures, the blank injection was repeated until carryover peaks were eliminated.

## RESULTS

**Fig. 2:**
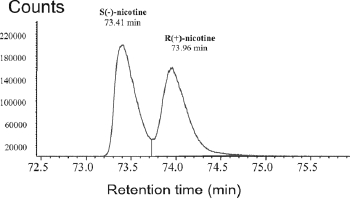
Chromatogram of R/S-nicotine (1.0 mg/ml), showing the separation of the two nicotine enantiomers following pyrolysis at 200°C.

As previously mentioned, the pyrolysate was separated using a chiral β-cyclodextrin (Cyclodex-B) column. This column was able to affect the enantioseparation of nicotine enantiomers. Figure 2 shows the resolution of R and S nicotine at 200°C. On a number of occasions, racemic nicotine was injected into the instrument to check that the enantioseparation had not deteriorated. Typically, the ratio of the peaks when integrated was determined as 51.1% (S) and 48.9% (R) showing that, in spite of the fact that the separation was sub-baseline, integration of the peaks remained broadly consistent with the expected ratio. The resolution factor was calculated as 1.0036.

**Fig. 3:**
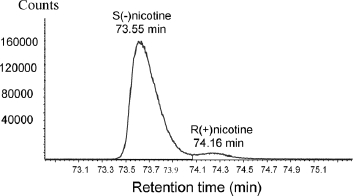
Chromatogram showing residual levels of (+)-(R)-nicotine present in (−)-(S)-nicotine (1.0 mg/ml) following pyrolysis at 200°C.

Figure 3 shows the chromatogram produced when (−)-(S)-nicotine was subjected to pyrolysis at 200°C. The minor peak (∼3%) at about 74.2 min was established as (+)-(R)-nicotine by comparing the retention time elution of (−)-(S)-nicotine and R/S-nicotine. The minor peak was apparent in all samples of (−)-(S)-nicotine examined, even when the pyrolysis temperature was as low as 200°C. The presence of small amounts of enantiomeric impurity in “pure” standards has been noted before—even when the sample did not encounter elevated temperatures when nicotine enantiomers were separated by HPLC.^5^ As mentioned previously, the enantiomeric purity of (−)-(S)-nicotine used in these experiments was 97–98%.

**Fig. 4:**
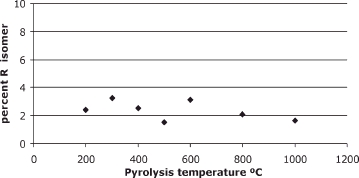
Graph showing the relationship of the percentage of (+)-(R)-nicotine as a proportion of total nicotine against temperature (mean of three replicates).

Our results show that a plot of the percentage of (+)-(R)-nicotine as a percentage of the total amount of nicotine is unchanged across the temperature range 200–1000°C indicating that pyrolytic racemization of nicotine does not occur (Fig. 4).

**Fig. 5:**
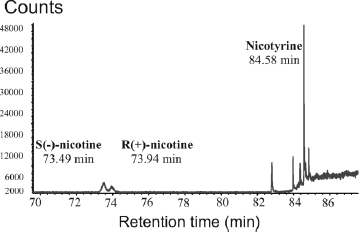
Chromatogram showing (−)-S and (+)-R-nicotine peaks and nicotyrine following the pyrolysis of R/S-nicotine (1.0 mg/ml) at 500°C.

**Fig. 6:**
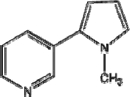
Molecular structure of β-nicotyrine.

When a sample of R/S-nicotine was injected, as shown in Figure 5, the three largest peaks eluted represent S and R nicotine, at 73.49 and 73.94 min, respectively, together with a compound that elutes around 84.58 min. This latter compound was identified as β-nicotyrine by comparing its mass spectrum with that referenced in the Wiley library. The structure of β-nicotyrine is shown in Figure 6. Nicotyrine is structurally similar to nicotine differing only in that the pyrrolidine ring has been aromatized by the removal of four hydrogen atoms and hence the elimination of the stereogenic centre. The thermal decomposition of nicotine to nicotyrine has been observed previously.^8^,^9^ Nicotyrine is considerably less basic than nicotine and almost certainly this accounts for its more favorable peak shape elution by GC.

Similarly, as observed for racemic nicotine, when (−)-(S)-nicotine (1.0 mg/ml) was subjected to pyrolysis GC, three peaks were identified in the chromatogram. These were: (−)-(S)-nicotine, (+)-(R)-nicotine (minor peak) and β-nicotyrine. Again β-nicotyrine was identified by comparing the MS spectra with the spectra present in the Wiley library of mass spectra. β-nicotyrine, boiling point 281°C, is expected to elute after nicotine. It is probable that the other peaks in the chromatogram are other breakdown products of nicotine, including further breakdown products of β-nicotyrine. There was also some evidence from MS spectra for the pyrosynthesis of myosmine (data not shown).

**Fig. 7:**
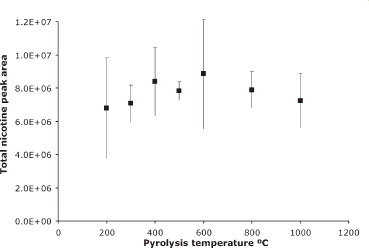
Peak area of total nicotine at different pyrolysis temperatures. Error bars show the 95% confidence interval of the mean of triplicate measurements.

**Fig. 8:**
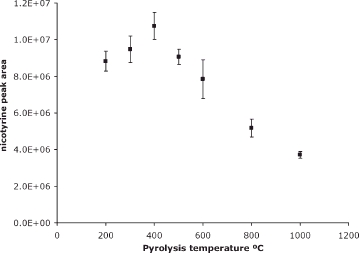
Peak area of nicotyrine at different pyrolysis temperatures. Error bars show the 95% confidence interval of the mean of triplicate measurements.

Using Agilent Chemstation software, the peak areas of the two nicotine enantiomers and nicotyrine were determined; plots of the peak areas of total nicotine and nicotyrine against temperature are shown in Figures 7 and 8, respectively.

From Figure 7, it may be seen that the total amount of nicotine (R and S) is stable following pyrolysis up to 1000°C. This is because under the conditions of open, online pyrolysis nicotine is able to volatilize away from the pyrolyser unit of the instrument (see Discussion).

Figure 8 shows the peak area of the nicotyrine peak following pyrolysis up to 1000°C. At the lower temperatures a general increase can be observed, reaching a maximum level at the pyrolysis temperature of 400°C, after which a progressive decrease follows. To eliminate the possibility that nicotyrine is already present in nicotine solutions, a purity check on the original (−)-(S)-nicotine sample was carried out on a separate (nonpyrolysis) GC/MS instrument. It was found that only trace amounts of nicotyrine (>0.1%) were present in the nicotine standard used in these experiments. Hence the high levels of nicotyrine found in the pyrolysis GC/MS originates from the pyrolysis of nicotine.

**Fig. 9:**
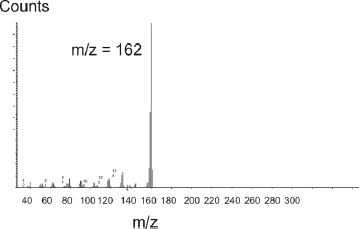
Mass spectrum of peak eluted at 84.5 min following pyrolysis of (2,4,5,6)-d_4_-R/S-nicotine (1.0 mg/ml) at 500°C.

To verify the observation that nicotyrine is a pyrolysis product of nicotine, a sample of isotopically labeled (2,4,5,6)-d_4_-R/S-nicotine was subjected to online pyrolysis GC/MS. When the labeled nicotine was subjected to online pyrolysis at 500°C a sharp peak was observed at 84.5 min, similar to Figure 5. The MS spectrum associated with this peak is shown in Figure 9 and shows a prominent signal at mass-to-charge ratio *(m/z)* of 162, the *m/z* ratio associated with d_4_-nicotyrine, which is 4 amu above the *m/z* of d_0_-nicotyrine. This confirms that nicotyrine is a pyrolysis product of nicotine.

## DISCUSSION

Our results show that a plot of the percentage of (+)-(R)-nicotine as a percentage of the total amount of nicotine is flat across the temperature range 200–1000°C indicating that pyrolytic autoracemization of nicotine does not occur (Fig. 4).

This is in contrast to the results presented by Liu et al.,^6^ who show a temperature-related increase in the pyrolytic racemization of (−)-(S)-nicotine solution with 21.8% of (+)-(R)-nicotine (as a portion of total nicotine) being observed at 550°C. Under our experimental conditions, the pyrolyser module was online with the GC/MS instrument and the pyrolysis time was 15 sec. However in the experiments conducted by Liu et al.,^6^ which showed synchronous increase of (+)-(R)-nicotine with temperature; pyrolysis was undertaken offline. In Liu's study pyrolysis involved a more lengthy exposure to high temperature, whereas in the experiments described here pyrolysis was online and was limited to 15 sec followed by immediate transfer and cooling to the GC inlet. The difference between online dynamic pyrolysis and static offline pyrolysis may explain the differences in outcome in the racemization observed. In offline pyrolysis, the components are trapped in a sealed chamber at high temperature for a significant period, whereas in online pyrolysis the components are flash vaporized and can be rapidly removed from the pyrolyser unit. Fournier et al.^8^ has reported that nicotine volatilizes below 250°C and so in online pyrolysis, nicotine is not expected to be exposed to high temperature in the pyrolyser unit for a significant time. The differences are experimental set-up and differences between open and closed pyrolysis almost certainly explains the divergence between our results and those of Liu et al.,^6^ who reports extensive racemization of nicotine following closed pyrolysis. Open pyrolysis is closer to the condition of a free burning cigarette.

Interestingly, when investigating the percentage amounts of (+)-(R)-nicotine present in main-stream and side-stream tobacco smoke Perfetti noted that the percentage of (+)-(R)-nicotine in side-stream smoke did not diminish in spite of the lower combustion temperatures associated with side-stream smoke.^4^ This is evidence that changes in pyrolysis temperature do not alter the ratio of (+)-(R)-nicotine to total nicotine in smoke.
